# Comparison of clinical outcomes with hip replacement versus PFNA in the treatment of intertrochanteric fractures in the elderly: A systematic review and meta-analysis (PRISMA)

**DOI:** 10.1097/MD.0000000000024166

**Published:** 2021-03-05

**Authors:** Junming Chen, Chen Yue, Peilin He, Zeling Huang, Li Li, Xue Zhang, Yanan Fan, Youwen Liu

**Affiliations:** a Key Laboratory of Orthopedics & Traumatology of Traditional Chinese Medicine and Rehabilitation (Fujian University of TCM), Ministry of Education, Fujian University of Traditional Chinese Medicine, Fuzhou; b Department of Orthopedic Surgery, Luoyang Orthopedic Hospital of Henan Province, Orthopedic Hospital of Henan Province, Luoyang, China.

**Keywords:** hip replacement, HR, intertrochanteric fractures, meta-analysis, PFNA, proximal femoral nail antirotation

## Abstract

**Background:**

The purpose of this meta-analysis was to evaluate the clinical efficacy and safety of HR and PFNA in the treatment of intertrochanteric fractures in the elderly.

**Methods:**

We carried out this review according to the principle of preferred reporting items for systematic reviews and meta-analyses (PRISMA) guideline. The clinical randomized controlled trials (RCTs), prospective cohort studies, retrospective cohort studies (RCSs), and case-control studies involving HR and PFNA in the treatment of intertrochanteric fractures in the elderly from 2000 to 2020 were compared by searching Web of Science, Pubmed, the Cochrane Library, and Embase. The quality of the included cohort study (CS) lines was evaluated using the Newcastle-Ottawa Scale (NOS). The quality of the included RCT lines was evaluated using Jadad. Forest plots were drawn by RevMan5.4 software based on the results and the data were analyzed.

**Results:**

After screening, a total of 9 articles were included, of which one was a clinical RCT and eight were RCSs with 1374 patients. The operative time of the PFNA group was shorter [WMD = 15.20; 95% CI (13.17, 17.23), *P* < .05] and the intraoperative blood loss was less [WMD = 178.81; 95% CI (97.24, 260.38), *P* < .05] than the HR group, while the first weight-bearing time of the HR group was shorter [WMD = −7.70; 95% CI (−10.54, −4.86), *P* *<* .05] than the PFNA group. There was no significant difference in the length of hospital stay, HHS, postoperative orthopedic complications, and postoperative medical complications between the 2 groups.

**Conclusion:**

With the development of HR technology and minimally invasive technology, the trauma caused by surgery is decreasing. Under the premise of improving perioperative management, such as optimizing the preoperative preparation and postoperative management, shortening the operative time, reducing intraoperative blood loss, and actively managing co-existing diseases, HR has more advantages than PFNA in the treatment of senile intertrochanteric fractures.

## 1 Introduction

An intertrochanteric fracture is a common type of hip fracture and is more common in the middle-aged and elderly.^[1]^ With the aging of the global population, the incidence of intertrochanteric fractures is also on the rise.^[2]^ Conservative treatment of intertrochanteric fractures requires patients to stay in bed for a long time, and many complications may occur, such as pneumonia, urethral infections, deep venous thromboses, and pressure sores. Indeed, it has been shown that the outcomes of conservative treatment are poor and the mortality rate is high.^[3,4]^ Currently, surgical treatment of intertrochanteric fractures is preferred.^[5,6]^ The main surgical methods include hip replacement (HR) and proximal femoral nail antirotation (PFNA). Because these surgical methods each have advantages and disadvantages, the choice between the 2 methods is controversial.^[7–9]^ More importantly, a meta-analysis comparing the efficacy of HR and PFNA has not been conducted. Therefore, this study will use the meta-analysis method to compare the corresponding indicators of HR and PFNA in the treatment of intertrochanteric fractures in the elderly, analyze these indicators to evaluate the clinical safety and effectiveness, and provide a new reference for clinical treatment strategies.

## 2 Methods

### 2.1 Search strategy

According to the preferred reporting items for systematic reviews and meta-analyses (PRISMA) principle of meta-analysis,^[10–12]^ we performed a literature search on Web of Science, Pubmed, The Cochrane Library, and Embase from 2000 to 2020 using the following key words: hip arthroplasty; hip replacement; total hip arthroplasty; total hip replacement; THA; HR; proximal femoral nail antirotation; PFNA; intertrochanteric fracture; and trochanteric femoral fracture. Studies focusing on HR and PFNA in the treatment of senile intertrochanteric fracture were also included. The search terms were connected with “OR” or “AND”.

### 2.2 Inclusion and exclusion criteria

The inclusion criteria were determined according to the PICOS principle, as follows:

1. design type clinical randomized controlled trial (RCT), prospective cohort study, retrospective cohort study (RCS), or case-control study;
2. age ≥ 60 years;
3. study subjects diagnosed with an intertrochanteric fracture; and
4. comparison of HR and PFNA.

The exclusion criteria were as follows:

1. the subjects had non-intertrochanteric fractures (including traumatic and pathological fractures);
2. intervention combined with a variety of treatment methods;
3. incomplete data or data that could not be obtained; and
4. repeated publication of the study.^[13]^

### 2.3 Literature screening and data extraction

The2 authors independently retrieved the titles and abstracts of articles, merged the literature retrieval results of the different databases, established an information database, and retained all eligible articles. The data of the 2 authors were extracted from the tables included in the trials and all the differences were unified through discussion.^[14–16]^ The following data were extracted from the included trials: first author; publication date; research topic; intervention; follow-up time; and results. The results included operative time, intraoperative blood loss, length of hospital stay, first weight-bearing time, postoperative Harris hip score (HHS), postoperative orthopedic complications, and postoperative medical complications. The design type and quality evaluation information of the studies were extracted.

### 2.4 Quality assessment

Two investigators independently employed the Newcastle-Ottawa scale (NOS) to assess the quality of the included cohort studies (CSs) by the method of selecting patients, the comparability of the study groups, and the study results. Based on these parameters, the total score was 9, with each study scoring a maximum of 1 on selection and results, 2 on comparability items, and greater than 6 on high-quality literature. The Jadad score was used to evaluate the quality of the included RCTs, including random sequence, allocation concealment, blind method, lost to follow-up and withdrawal, in which 0 to 3 represents low-quality studies and 4 to 7 represents high-quality articles. When the opinions could not be unified, a third party participated in the evaluation.^[17]^

### 2.5 Outcome indicators

The operative time, intraoperative blood loss, length of hospital stay, first weight-bearing time, postoperative HHS, postoperative orthopedic complications, and postoperative medical complications were recorded. The main outcome indicators were operative time, intraoperative blood loss, and the first weight-bearing time. The secondary outcome indicators were postoperative HHS, length of hospital stay, postoperative orthopedic complications, and postoperative medical complications.^[18]^

### 2.6 Evidence quality and recommendation level

According to the results of the system evaluation, the GRADE system recommendation method was used to evaluate the quality of evidence and recommendation grade. The quality grade was divided into the following 4 grades: very low; low; medium; and high, and edited and analyzed by GRADEpro3.6.1 software.

### 2.7 Statistical analysis

The Cochrane Collaboration Network Revman5.4 software (https://www.cochrane.org/) was used for our meta-analysis. Before the analysis of each study, a χ^*2*^ test was used to determine whether there was heterogeneity between studies. When no heterogeneity existed between studies (*P* *>* .1, *I*^*2*^ *<* 50%), a fixed effect model was used. When there was heterogeneity between studies (*P* *<* .1, *I*^*2*^ > 50%), the causes of heterogeneity were analyzed first, then sensitivity analysis was carried out. If there was clinical heterogeneity, a subgroup analysis was needed, and if there was still heterogeneity, a random effect model or descriptive assessment was used. The counting data were analyzed by RR or OR, continuous variable data by MD analysis, and the 95% CI was calculated. Forest plots were used to display the results of the meta-analysis.

## 3 Results

### 3.1 Literature retrieval results

**Figure 1:**
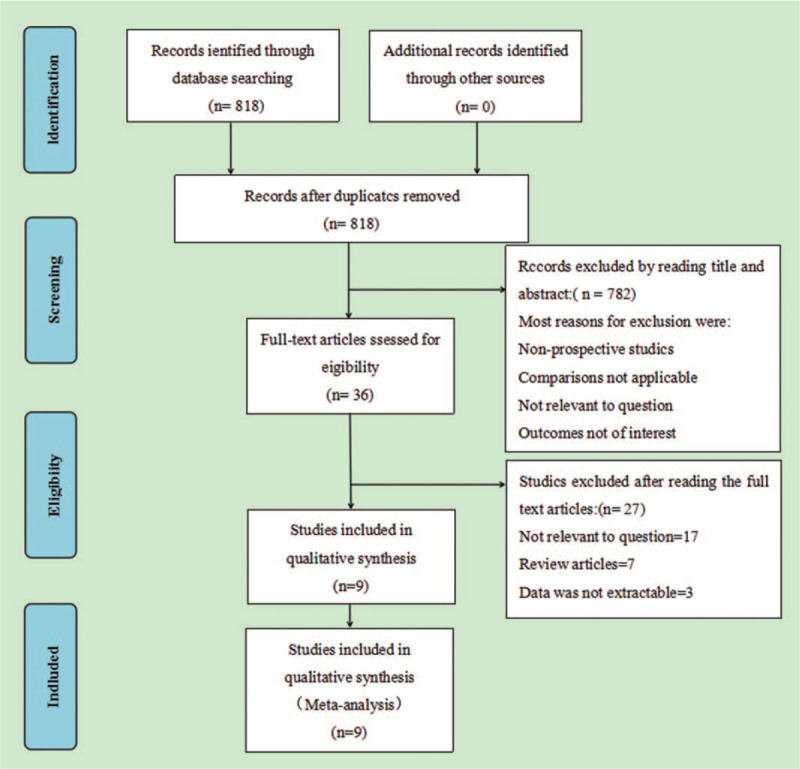
Search results and selection procedure.

A total of 818 articles were retrieved. The abstracts were read carefully, 782 articles were excluded, and 36 full-text articles were downloaded. According to the inclusion and exclusion criteria, 27 articles that did not meet the inclusion criteria during the second screening, thus 9 articles involving 1374 patients were included (Fig. 1).

### 3.2 Study quality evaluation

The included studies included 1 RCT and eight CSs. The results of the NOS showed that eight and 7 studies met the criteria for patient selection and comparability between groups, respectively; however, none of the studies had complete outcome reporting. The NOS scores of 8 studies were ≥6. The Jadad score of 1 study was ≥4, as shown in Table 1.

**Table 1:** Characteristics of the included studies.

Study	Year	Country	Outcome indicators	Age	Sample size	HR	PFNA	Adverse event	Study type	NOS/Jadad
Esen et al ^[19]^	2017	Turkey	1/6/7	≥60	92	58	34	No	CS	7
Görmeli et al ^[20]^	2015	Turkey	1/2/5	>65	143	75	68	No	CS	7
Li et al ^[21]^	2019	China	1/2/5/6/7	≥60	97	46	51	No	CS	8
Luo et al ^[22]^	2017	America	1/2/3/4/5/6/7	≥70	123	52	71	No	CS	8
Tang et al ^[23]^	2012	Netherlands	5/6/7	≥65	303	156	134	No	CS	7
Xie et al ^[24]^	2019	Belgium	1/2/3/4/5/6	≥60	367	172	195	No	CS	7
Suh et al ^[25]^	2015	South Korea	5	≥70	100	50	50	No	CS	6
Zhou et al ^[26]^	2019	England	1/2/3/5/6/7	≥75	108	47	61	No	CS	7
Özkayin et al^[27]^	2015	Netherlands	1/5/6/7	≥75	54	33	21	No	RCT	4

### 3.3 Meta-analysis results

#### 3.3.1 Operative time

**Figure 2:**
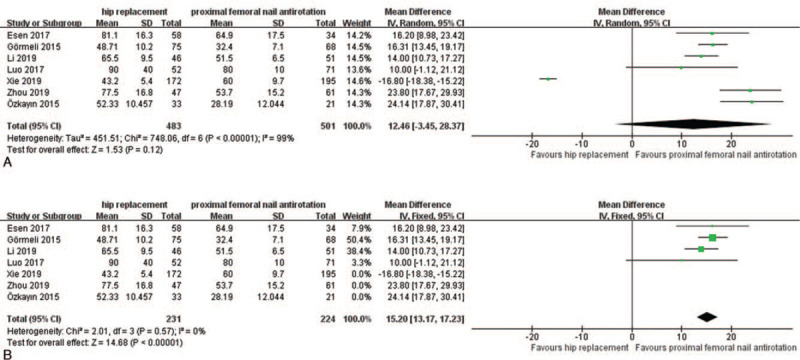
A. A forest plot comparing the HR and PFNA operative times. B. A forest plot comparing the HR and PFNA operative times after excluding studies caused heterogeneity.

Seven articles were included in the study to compare the mean operative time between the HR and PFNA groups. Of the 984 patients, 483 were in the HR group and 501 were in the PFNA group. The results of the heterogeneity analysis showed (*P* = .12, *I*^*2*^ = 99%) that there was heterogeneity among the studies (Fig. 2A), which was due to some of the studies (Xie2019, Zhou2019, Özkayin2015). After removing studies, the meta-analysis was repeated and the results of the heterogeneity analysis showed were an indication for the fixed effect model analysis method (*P* < .0001, *I*^*2*^ = 0%), and the data analysis [WMD = 15.20; 95% CI (13.17, 17.23), *P* < .0001] is shown in Figure 2B (*P* < .05). The average operative time in the PFNA group was shorter than the HR group.

#### 3.3.2 Intraoperative blood loss

**Figure 3:**
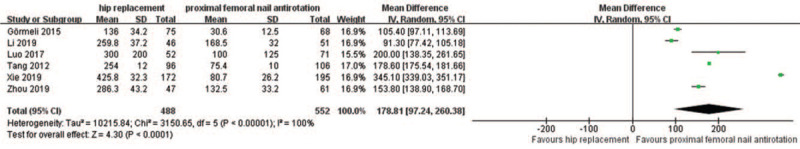
A forest plot for comparing HR and PFNA intraoperative blood loss.

Six articles were included to compare the mean intraoperative blood loss between the HR and PFNA groups, involving 1040 patients (488 patients in the HR group and 552 patients in the PFNA group). The results of the heterogeneity analysis showed that there was significant heterogeneity among the studies (*P* < .0001, *I*^*2*^ = 100%); the heterogeneity could not be eliminated after sensitivity and subgroup analyses, thus the random effect model analysis method was used. As shown in Figure 3 (*P* < .05) data analysis revealed the following: [WMD = 178.81; 95% CI (97.24, 260.38), *P* < .0001]. The average intraoperative blood loss in the PFNA group was less than the HR group.

#### 3.3.3 Length of hospital stay

**Figure 4:**
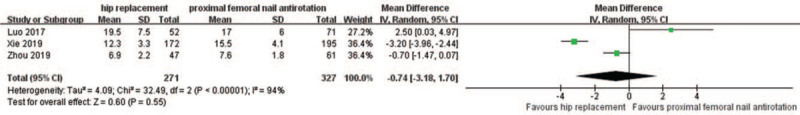
A forest plot comparing HR and PFNA length of hospital stay.

Three articles were included to compare the average length of hospital stay between the HR and PFNA groups. Of 594 patients, 271 were in the HR group and 323 were in the PFNA group. The results of the heterogeneity analysis showed that heterogeneity existed among studies (*P* < .00001, *I*^*2*^ = 94%) that could not be eliminated after sensitivity and subgroup analyses. The random effect model analysis method was used. Data analysis showed the following, as shown in Figure 4 (*P* > .05): [WMD = −0.74; 95% CI (−3.18, 1.70), *P* = .55]. There was no significant difference in the average length of hospital stay between the PFNA and HR groups.

#### 3.3.4 First weight-bearing time

**Figure 5:**
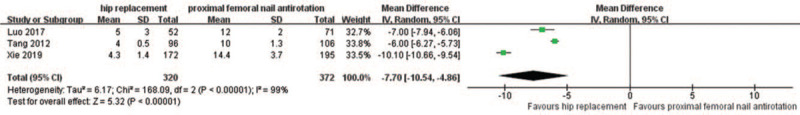
A forest plot comparing HR and PFNA time to first weight-beariing.

A total of 3 articles were included, and the first weight-bearing time of the HR and PFNA groups was determined. Of the 692 patients, 320 were in the HR group and 372 were in the PFNA group. The results of the heterogeneity analysis showed that heterogeneity existed among the studies (*P* < .00001, *I*^*2*^ = 99%) that could not be eliminated after sensitivity and subgroup analyses. The random effect model analysis method was used. The results of data analysis showed the following, as shown in Figure 5 (*P* < .05): [WMD = −7.70; 95% CI (−10.54, −4.86), *P* *<* .00001]. The first weight-bearing time of the HR group was shorter than the PFNA group.

#### 3.3.5 Postoperative HHS

**Figure 6:**
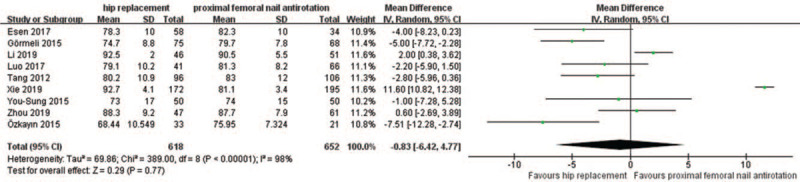
A forest plot comparing HR and PFNA postoperative HHS.

Nine articles were included to analyze HHS after surgery in the HR and PFNA groups. Of the 1270 patients, 618 were in the HR group and 652 were in the PFNA group. The results of the heterogeneity analysis showed that there was heterogeneity among studies (*P* < .00001, *I*^*2*^ = 98%), but the heterogeneity could not be eliminated after sensitivity and subgroup analyses. Thus, the random effect model analysis method was selected. Data analysis showed the following, as shown in Figure 6: [WMD = −0.83; 95% CI (−6.42, 4.77), *P* = .77]. There was no significant difference in hip function between the HR and PFNA groups after surgery.

#### 3.3.6 Postoperative orthopedic complications

**Figure 7:**
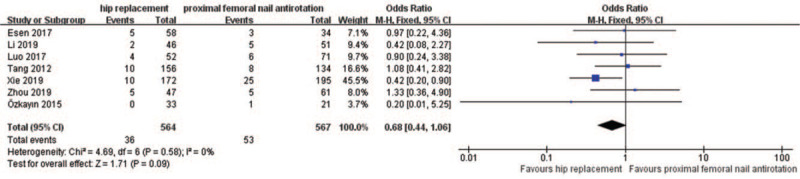
A forest plot comparing HR and PFNA postoperative orthopedic complications.

A total of 7 studies recorded postoperative orthopedic complications in the HR and PFNA groups, and included 1131 patients, with 564 in the HR group and 567 in the PFNA group. The results of the heterogeneity analysis showed that the studies were homogeneous (*P* = .58, *I*^*2*^ = 0%), thus the fixed effect model analysis method was used. Data analysis showed the following, as shown in Figure 7: [OR = 0.68; 95% CI (0.44, 1.06), *P* = .09], was not significant. There was no significant difference in the incidence of postoperative orthopedic complications between the HR and PFNA groups.

#### 3.3.7 Postoperative medical complications

**Figure 8:**
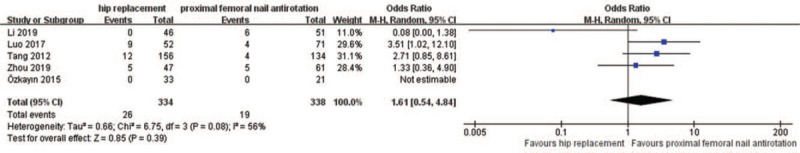
A forest plot comparing HR and PFNA postoperative medical complications.

A total of 5 studies recorded the postoperative medical complications in the HR and PFNA groups. Of the 672 patients, 334 were in the HR group and 338 were in the PFNA group. The results of the heterogeneity analysis showed that there was heterogeneity among the studies (*P* = .08, *I*^*2*^ = 56%) and that the heterogeneity could not be eliminated after sensitivity and subgroup analyses, so the random effect model analysis method was selected. Data analysis showed the following, as shown in Figure 8: [OR = 1.61; 95% CI (0.54, 4.84), *P* = .39]. There was no significant difference in the incidence of post-operative medical complications between the HR and PFNA groups.

### 3.4 Sensitivity analysis and publication bias

Due to the limited number of articles (n = 9), neither a funnel plot nor Egger test was generated to detect potential publication bias; no publication bias was noted.

### 3.5 Results of the GRADE evaluation and recommended intensity of indicators

GRADEpro3.6.1 software was used to evaluate the quality of evidence for 3 key outcome indicators and 4 secondary outcome indicators. Although the results of the bias risk assessment section showed that the quality of the trial design was reasonable. The summary of the results showed that the overall level of evidence available in our meta-analysis was medium or low, which may be due to inconsistencies, especially with respect to the limited number of events (Table 2).

**Table 2:** Outcome indicator of the GRADE evidence quality evaluation results.

Continuous variable compared to placebo for intertrochanteric fractureBibliography: Hip replacement vs proximal femoral nail antirotation for intertrochanteric fracture. Cochrane Database of Systematic Reviews [Year], Issue [Issue].											
Certainty assessment							Summary of findings				
							Study event rates (%)			Anticipated absolute effects	
Participants (studies) Follow up	Risk of bias	Inconsistency	Indirectness	Imprecision	Publication bias	vJVcrcill certainty of evidence	With placebo	With Continuous variable	Relative effect (95% CI)	Risk with placebo	Risk difference with Continuous variable
Operation time											
984 (7 observational studies)	not serious	not serious	not serious	not serious	none	LOW	501	483	–	The mean operation time was 0	MD 12.46 higher (3.45 lower to 28.37 higher)
Intraoperative bleeding volume											
1040 (6 observational studies)	not serious	not serious	not serious	not serious	none	LOW	552	488	–	The mean intraoperative bleeding volume was 0	MD 178.81 higher (97.24 higher to 260.38 higher)
Hospitalization time											
598 (3 observational studies)	not serious	serious	serious	serious	none	VERY LOW	327	271	–	The mean hospitalization time was 0	MD 0.74 lower (3.18 lower to 1.7 higher)
First weight-bearing time											
692 (3 observational studies)	not serious	not serious	not serious	not serious	none	LOW	372	320	–	The mean first weight bearing time was 0	MD 7.7 lower (10.54 lower to 4.86 lower)
Postoperative Harris hip score											
1270 (9. observational studies)	not serious	serious	serious	serious	none	VERY LOW	652	618	–	The mean postoperative Harris hip score was 0	MD 7.15 higher (6.53 higher to 7.78 higher)

Binary variable compared to placebo for intertrochanteric fractureBibliography: Hip replacement vs proximal femoral nail anti rotation for intertrochanteric fracture. Cochrane Database of Systematic Reviews [Year], Issue [Issue].											
Certainty assessment							Summary of findings				
							Study event rates (%)			Anticipated absolute effects	
Participants (studies) Follow up	Risk of bias	Inconsistency	Indirectness	Imprecision	Publication bias	Overall certainty of evidence	With placebo	With Binary variable	Relative effect (95% CI)	Risk with placebo	Risk difference with Binary variable
Postoperative orthopedic complications											
733 (5 observational studies)	serious	serious	serious	serious	none	VERY LOW	40/372 (10.8%)	21/361 (5.8%)	OR 0.52 (0.30 to 0.91)	108 per 1,000	49 fewer per 1000 (from 73 fewer to 9 fewer)
Postoperative medical complications											
672 (5 observational studies)	serious	serious	serious	serious	none	VERY LOW	19/338 (5.6%)	26/334 (7.8%)	OR 1.61 (0.54 to 4.84)	56 per 1,000	31 more per 1000 (from 25 fewer to 168 more)

## 4 Discussion

Intertrochanteric fractures are more common in the middle-aged and elderly. Because osteoporosis is common in the elderly, intertrochanteric fractures are more likely to occur in cases involving external force collisions and violence.^[28]^ The choice of surgical treatment enables patients to carry out weight-bearing exercise as soon as possible, speed up functional recovery of the lower limbs, avoid the occurrence of a hip varus deformity after fracture, improve the quality of life, and increase survival of postoperative patients.^[29]^ At present, HR technology is more mature, the prosthesis is firm and stable, and postoperative hip joint function recovers earlier.^[30–31]^ Although HR has obvious advantages, the operative time of HR is longer, the amount of intraoperative blood loss is more, and the degree of tissue injury is also greater. For some elderly patients, due to their weak physical level, they have a low tolerance for surgery, which will be accompanied by a series of risk factors. At the same time, the service life of the prosthesis is limited, so there is still controversy with respect to HR.^[22]^ PFNA was developed based on the improvement of internal fixation technology. The operative time is short, the femoral head and neck can be preserved, and the fixation effect is good. The characteristics of PFNA include the following: avoid the instability of the proximal femur after internal fixation; conducive to fracture healing; and reduce the incidence of hip varus deformity.^[32]^ Zhou et al^[26]^ reported that hip joint function of patients is effectively improved after surgical treatment, which significantly reduces the incidence of post-operative complications. Based on the above advantages, PFNA is suitable for elderly patients with partial intertrochanteric fractures because this treatment requires low surgical tolerance.^[33]^ The literature shows that there is a serious risk of fixation failure and loosening in the clinic. The PFNA failure rate of patients with severe osteoporosis is higher, and the mortality rate 1 year postoperative can reach 21.4%, which may be related to the longer time in bed postoperatively and delay in weight-bearing.^[34]^

HR and PFNA are the 2 main surgical methods for the treatment of femoral intertrochanteric fractures. Because there are significant differences in the efficacy and prognosis of the 2 surgical methods for different patients, the indications for elderly patients with intertrochanteric fracture should be combined with the clinical characteristics on the premise of strengthening perioperative treatment, considering the patients physical condition, fracture, and severity of the injury. Strengthening perioperative management includes preoperative preparation and postoperative treatment, improving surgical skills, reducing intraoperative blood loss, actively treating basic diseases, correcting anemia, and treating complications.

A total of 9 articles were included in this meta-analysis, and the total number of cases was relatively small. The quality of some literature research was not high, and the literature evaluation criteria were not unified. These factors will increase the difficulty of arriving at a meaningful conclusion, so it is necessary to establish and select unified measurement standards and functional evaluation standards in clinical research.

## 5 Conclusion

The appropriate treatment scheme should be chosen for elderly patients with intertrochanteric fractures. HR promoted the functional recovery of the hip joint, shortened the time of bed rest, and enabled patients to carry out weight-bearing exercise in the early stage. With the development of HR and minimally invasive technology, the trauma caused by surgery was getting smaller and smaller. Under the premise of strengthening perioperative management, HR had more advantages in the treatment of femoral intertrochanteric fractures in the elderly. For patients with severe osteoporosis and other diseases, it was necessary to make adequate preoperative preparation and postoperative treatment, multidisciplinary consultation, and active treatment of basic diseases, thus giving patients the most scientific diagnosis and treatment plan and reducing the occurrence of postoperative complications. Only in this way can patients achieve satisfactory results and recover their health as soon as possible.

## Author contributions

**Conceptualization:** Junming Chen, Liu Youwen.

**Data curation:** Chen Yue, Zeling Huang, Li Li, Xue Zhang, Yanan Fan.

**Formal analysis:** Chen Yue, Peilin He.

**Funding acquisition:** Peilin He.

**Methodology:** Chen Yue.

**Project administration:** Chen Yue.

**Supervision:** Liu Youwen.

**Writing – original draft**: Junming Chen, Peilin He.

**Writing – review & editing:** Chen Yue, Peilin He, Zeling Huang, Li Li, Xue Zhang, Yanan Fan, Liu Youwen.
